# The emotional profile of Slovak freshmen in teaching English as a foreign language

**DOI:** 10.3389/fpsyg.2024.1410467

**Published:** 2024-10-11

**Authors:** Zdena Kralova, Frantisek Petrovic, Karla Hrbackova, Jessica Sebokova

**Affiliations:** ^1^Faculty of Education, Constantine the Philosopher University in Nitra, Nitra, Slovakia; ^2^Faculty of Humanities, Tomas Bata University, Zlin, Czechia; ^3^Faculty of Natural Sciences and Informatics, Constantine the Philosopher University in Nitra, Nitra, Slovakia

**Keywords:** student teachers, English, freshmen, emotions, triggers

## Abstract

The article focuses on tracing changes in Slovak pre-service TEFL student teachers’ emotional states over the first semester of their university study and detecting possible factors inciting their emotions. It highlights the importance of understanding these emotional states as they significantly impact students’ perceptions of their suitability as educators. A mixed-methods approach was employed to collect qualitative and quantitative data through questionnaires, reflections, and interviews. The sample consisted of 67 non-native Slovak first-year TEFL students. It was found that the students experienced a range of emotions, from initial confusion and worry to eventual joy and contentment. Negative emotions were mainly triggered by the flood of new information and study-related factors, while positive emotions were associated with the supportive attitude of teachers and the freedom of university life.

## 1 Introduction

Teaching is undeniably a profession that is deeply intertwined with emotions (Nguyen, 2019). This is particularly true in language instruction, where the teacher’s personality traits and emotional states can significantly influence their ability to effectively communicate with students (Pianta and Allen, 2008). Krashen’s (1982) affective filter hypothesis sheds light on the emotional factors affecting language learning. When learners experience anxiety, their affective filter may rise, hindering effective language learning. In the context of foreign language teacher education, understanding and addressing these affective components become essential.

Learning to teach has long been viewed as a cognitive process (Korucu Kış, 2021). Developing pre-service teachers’ understanding of the subject, pedagogy, and foundations of education is the goal of typical language teacher education programs (Shulman, 1987). Lemarchand-Chauvin and Tardieu (2018) also point out that the majority of teacher education programs have long ignored the emotive component of teaching in favor of professional and didactic training.

However, with the rise of sociocultural perspectives in teacher education (Tripon, 2022), it has become clear that learning to teach entails more than just acquiring and expressing subject matter; it also entails learning how to handle the affective components of teaching (Martínez Agudo, 2018). Thus, it appears that the widely accepted definition of teacher cognition (Borg, 2003) has to be revised to incorporate emotions as a crucial component of professional development, according to recent research (e.g., Golombek and Doran, 2014). In the post-pandemic era, this has become an even more pressing need, as highlighted by some authors (Martínez-Líbano et al., 2023).

The number of research articles on the emotions of pre-service teachers has increased over the last decade (Korucu Kış, 2021). Pre-service teachers’ feelings regarding their teaching practicum have been the subject of most of these studies (e.g., Teng, 2017; Timostsuk and Ugaste, 2012), although the course of the study remains rather under-investigated. One of the few, Oyama (2021), examined the relationship between foreign language enjoyment, self-efficacy, engagement, and L2 vocabulary learning among Japanese first-year university students of English over one semester.

During their initial teacher education coursework, student teachers go through a range of emotions, including anger, anxiety, disappointment, doubt, irritation, fear, love, enthusiasm, satisfaction, and pride (Teng, 2017). Martínez-Líbano and Yeomans-Cabrera (2023) identified several factors (including depressive symptoms and low parental support) that influence the risk of dropping out of the Chilean higher education system. Psychosocial variables may have a major impact not only on students’ learning of professional knowledge and pedagogical skills but also on their understanding of and commitment to the teaching profession (Yuan and Lee, 2015).

The research problem the current study addresses is the changes and factors of emotional states in Slovak first-year university students studying to become teachers of English as a foreign language. These students are at the beginning of their journey into the teaching profession, and their emotional states during this critical period may have a profound impact on their professional development. What is more, this is the time when many lose motivation and drop out. According to available data, about one third of students in OECD and EU countries leave higher education without a degree (OECD, 2013). In Slovakia, 33% of students most frequently drop out in the first year of a full-time bachelor’s degree program (MSVVS SR, 2017).

## 2 Materials and methods

### 2.1 Objectives

The primary objective of the current longitudinal study was to trace changes in Slovak pre-service TEFL student teachers’ emotional states over the first semester and to detect possible factors inciting their emotions. The mixed model design was utilized and both quantitative and qualitative data were collected by questionnaires, reflections, and interviews.

The following research questions were formulated:

1. What are the changes in participants’ emotional profiles during their 1st semester?
2. What are the triggers of these changes?

### 2.2 Sample

A total of 67 Slovak first-year university students of TEFL, who voluntarily agreed to participate in the research, served as a sample in this study. They were selected by convenience sampling and shared several additional variables, such as their level of English proficiency (B2) and age (18–19 years). The inclusion criteria for participants included being a first-year TEFL student at the given university and providing written informed consent. The exclusion criteria were incomplete or missing data from the questionnaires and reflections. The study was conducted under the authorization of the Ethics Committee of Constantine the Philosopher University in Nitra under resolution no. UKF/74/2024/191013:003.

### 2.3 Instruments

Related studies have mostly used semi-structured interviews (Hanna et al., 2019) and validated questionnaires (e.g., Martínez-Líbano and Yeomans, 2023) to provide an understanding of individuals’ emotional experiences. To the best of our knowledge, only Chen et al. (2022), who studied how student teachers’ emotions interacted with their professional identities, drew data from the triangulation of methods. The current study hypothesized that students’ emotional states change significantly during their first semester and that these changes are primarily triggered by study-related factors. To test these hypotheses, data collection methods including questionnaires, reflections, and group interviews were adopted.

#### 2.3.1 Questionnaire

The Brunel Mood Scale (BRUMS) (Terry and Lane, 2000) is one of the most widely used standardized self-report measures for capturing the emotional profile of an individual. It consists of 24 mood descriptor adjectives divided into six subscales: vigor, confusion, depression, anger, tension, and fatigue. Responses were given on a 5-point Likert scale ranging from 0 (not at all) to 4 (extremely), with higher scores indicating higher levels of emotion.

#### 2.3.2 Reflections

Participants were suggested to reflect on their emotional states at the beginning and the end of the 1st semester, which aimed to elicit the emotions they experienced and their triggers. Two open questions were asked: *How do you feel at the beginning/end of your first semester? What are the reasons for such feeling*s? All reflection sheets were written in the participants’ native language.

#### 2.3.3 Interview

A semi-structured group interview was used in conjunction with the reflections to go deeper into the participants’ self-perceptions of their emotional state. First, the research objective was transformed into two direct-form questions: *What emotional changes have you experienced during the 1st semester of your university study? What has led to these changes?* The interviewer also considered some prompts to clarify the questions and probes to ask participants to provide details.

The interview was conducted in the participants’ native language during the last week of the first semester. The interviewer (one of the authors—a sophomore TEFL student) informed the participants about the purpose and conduct of the interview and asked for permission to record the responses.

### 2.4 Data analysis

#### 2.4.1 Questionnaire

The timeframe “How do you feel these days?” was used, and the students completed the questionnaire during the first and the penultimate week of their first semester. The current study used the verified Czech adaptation of BRUMS (Kveton et al., 2020) translated into Slovak (𝜔 > 0.70). Repeated measures ANOVA through IBM SPSS 29 was made to determine changes in scores from pre-test to post-test, which were analyzed using descriptive statistics.

#### 2.4.2 Reflections

The combined approach to coding was applied to data sets (Saldana, 2009). Deductive coding was used for the emotions. Parrott (2002) tree classification of emotions, with primary emotions (love, joy, surprise, anger, sadness, and fear) further subdivided into secondary and tertiary emotions, was identified as the basis for coding.

The reflection sheets were subjected to manual coding. Two of the authors read through the data and assigned excerpts to the codes – primary emotions (for example: *“I’m afraid I will not understand”*—fear). In the next round, they were refined and assigned to the secondary (e.g., nervousness) and tertiary (e.g., worry) emotions by another researcher. The classification was then reviewed by a psychologist to increase the validity of the results. One code (confusion) was added because new ideas emerged during the coding process that were not captured by the existing codes. However, confusion is defined as a negative emotion in several other classifications of emotions.

For triggers, inductive coding was used and the codes were derived from the data. The first round of coding was done by two authors who independently coded the data *in vivo*, summarizing participants’ words into the list of initial codes (for example: *“Departmental websites are so outdated”*—lack of information). The coding was then discussed with the first researcher, and two basic themes were developed. The codes were then combined into the themes: study-related triggers (study, information, language, profession, teachers, and schedule) and study-unrelated triggers (peers, freedom). Finally, the tertiary emotions were divided into positive and negative ones, paired with their associated triggers, and organized for comparison over time.

#### 2.4.3 Interview

The interviews were transcribed via Sonix (Sonix, Inc, 2024), and the transcripts were subjected to manual coding. Given the research questions and identical objective categories in both data sets—emotions and their triggers—a mixed coding strategy as described in Section 2.4.1 was applied to the interview coding.

### 2.5 Procedure

**Figure 1:**
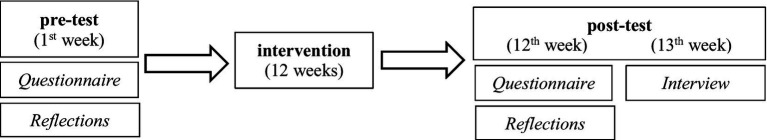
Summary of the research design.

The study was conducted in three stages—pre-test (the first week of the 1st semester), intervention (12 weeks of the 1st semester), and post-test (the penultimate and the last week of the 1st semester) as shown in Figure 1. In the pre-test and post-test, the participants filled in the questionnaire and answered the reflection questions. During the last week of the semester, an interview was performed with all the students randomly divided into three groups.

## 3 Results

### 3.1 Questionnaire

Although the mean scores for confusion and tension decreased in the post-test, the difference between the pre-test and post-test scores was not statistically significant in any of the emotional subscales (Table 1).

**Table 1:** The Brunel mood scale results.

	Intervention	Comparison	*F*	*p*	Effect size
Vigor					
pre-test	6.9 (2.81)	6.56 (3.29)			
Post-test	6.62 (3.17)	6.78 (3.92)	0.183	0.671	0.004
Change	−0.29	0.22			
Confusion					
Pre-test	4.86 (3.67)	6.59 (3.66)			
Post-test	2.14 (2.92)	3.22 (2.72)	0.436	0.512	0.009
Change	−2.71	−3.37			
Depression					
Pre-test	2.48 (3.64)	3.63 (3.66)			
Post-test	1.86 (3.05)	2.37 (2.71)	0.342	0.562	0.007
Change	−0.62	−1.26			
Anger					
Pre-test	0.48 (0.87)	1.81 (2.59)			
Post-test	0.71 (1.31)	1.89 (2.33)	0.933	0.339	0.020
Change	0.24	0.07			
Tension					
Pre-test	5.24 (4.61)	4.67 (3.57)			
Post-test	3.57 (3.20)	3.04 (2.88)	0.001	0.970	0.001
Change	−1.67	−1.63			
Fatigue					
Pre-test	3.76 (2.97)	6.70 (3.37)			
Post-test	4.62 (2.36)	5.81 (3.49)	2.115	0.153	0.044
Change	0.86	−0.89			

### 3.2 Reflections

As shown in Table 2, most participants reported positive emotions of excitement (*N* = 60/67) and thrill (*N* = 51/67) at entry into the university study. They also shared negative feelings of confusion (*N* = 56/67), worry (*N* = 48/67), and nervousness (*N* = 33/67). At the exit of their first semester, they generally experienced positive emotions—mostly contentment (*N* = 48/67), relief (*N* = 32/67), excitement (*N* = 28/67), and joy (*N* = 27/67). However, apart from these positive feelings, negative emotions were also reported. Almost all participants reported annoyance (*N* = 62/67), despair (*N* = 35/67), and frustration (*N* = 23/67).

**Table 2:** The integration of qualitative data.

Research question	Reflections				Interview			
	September		December		September		December	
	Positive	Negative	Positive	Negative	Positive	Negative	Positive	Negative
Emotion (trigger)	Excitement (peers) Thrill (study)	Confusion (information) Worry (study) Nervousness (teachers)	Contentment (teachers) Relief (study) Excitement (freedom) Joy (peers)	Annoyance (schedule) Despair (schedule) Frustration (information)	Enthusiasm (language) Thrill (profession)	Confusion (information) Anxiety (language)	Joy (language) Elation (profession)	Fear (study) Worry (profession)

### 3.3 Interview

The students’ initial enthusiasm and thrill turned into joy and elation. In the beginning, many participants felt confused and anxious, and toward the end of the semester, some reported fear and worry.

## 4 Discussion

The study aimed to trace changes in the emotional states of Slovak pre-service TEFL student teachers during their first semester and to identify the factors influencing these emotions. Key findings indicate that students initially experienced negative emotions, mainly due to the influx of new information. However, over time, positive emotions became more prevalent, attributed to supportive teachers and the freedom of university life. These results align with the study’s objectives by highlighting the importance of understanding emotional states in teacher education programs as emotions significantly impact students’ perceptions of their suitability as educators. This study contributes to the broader field by highlighting the role of emotional factors in teacher training, suggesting that teacher education programs should incorporate emotional support strategies to enhance student development (Kralova and Tirpakova, 2019).

The study revealed several key results. Initially, most students reported confusion and worry, primarily triggered by overwhelming new information and academic challenges (*“I was told that I would be just a number at a university”*). On the other hand, they were excited about meeting new people and enthusiastic about their chosen field of study. As the semester progressed, there was a noticeable shift toward joy and contentment, largely due to the supportive attitude of teachers (*“It is very nice of them to address us by our first names”*), and the increased autonomy. Additionally, it was found that despite a general trend toward positive emotions, frustration and annoyance remained due to organizational issues such as overlapping and late class schedules and insufficient information. Though the statistical analysis revealed no significant changes in the emotional states, the mean values for confusion and tension decreased considerably in the post-test, which is consistent with the data from the qualitative research instruments.

The results reveal a complex emotional trajectory that is both consistent with and contradictory to previous research. Initially, students experienced negative emotions echoing the findings of Nguyen (2019) and Teng (2017) that the beginning of university study is often marked by high levels of anxiety among students. However, the shift toward positive emotions parallels Oyama’s (2021) findings and Krashen’s (1982) Affective Filter Hypothesis which posit that a supportive learning environment can lower anxiety and enhance learning. Contrary to some studies (Martínez-Líbano and Yeomans, 2023; Yang and Koo, 2022), which emphasized personal and social factors as primary emotional triggers, this study identified academic-related factors as more critical. This discrepancy could be due to the specific context of Slovak higher education, where institutional support and teacher-student interactions may play a more prominent role. Overall, while the study’s results mostly align with existing literature, the emphasis on academic over personal/social factors presents a unique perspective that warrants further exploration in diverse educational contexts.

Furthermore, while Hall et al. (2019) emphasized the significance of institutional support, the present study stresses the need for better administrative coordination to address ongoing frustrations related to class schedules and information dissemination. These differences might also stem from varying levels of student autonomy, with Slovak students possibly experiencing a distinct balance between independence and support compared to their counterparts in other regions. The findings contribute to the current understanding of teacher education by highlighting the importance of addressing both emotional and organizational factors to enhance student wellbeing and retention. The analysis emphasizes the need for a nuanced approach that considers cultural and contextual variations, thereby advancing the field by encouraging more tailored support strategies in diverse educational settings.

The findings have significant practical implications for educators, policymakers, and other stakeholders. For educators, the results underscore the importance of creating a supportive learning environment to facilitate positive emotional experiences. Teachers should be trained to recognize and address students’ emotional needs, incorporating strategies such as personalized feedback and fostering a collaborative classroom atmosphere. For instance, providing timely and clear information about course expectations and offering individualized support can alleviate students’ initial confusion and anxiety. Policymakers can use these insights to enhance teacher education programs by integrating emotional intelligence training and support mechanisms for pre-service teachers. Establishing mentorship programs where experienced educators guide new students through their initial transition period could be particularly beneficial.

Additionally, educational institutions should improve administrative coordination to avoid scheduling conflicts and ensure timely communication of important information, thus reducing students’ frustration. These measures can help lower dropout rates and improve overall student satisfaction and retention. Stakeholders should also consider implementing regular feedback sessions to monitor students’ emotional wellbeing throughout their studies. By addressing both the academic and emotional aspects of teacher education, the findings contribute to a more holistic approach to developing competent and resilient educators, ultimately enhancing the quality of education in the broader context.

The study acknowledges several limitations that might have impacted the results. Firstly, the sample size was relatively small and limited to a specific cultural and educational context, which may affect the generalizability of the findings. The convenience sampling method used could also introduce selection bias, as the participants who volunteered may not fully represent the broader population of TEFL students. Additionally, the study’s reliance on self-reported data through questionnaires and reflections may be subject to response bias, where students might have underreported or over reported their emotional states.

To address these limitations, future research should aim for a larger and more diverse sample, encompassing different cultural and educational contexts to enhance the external validity of the findings. Longitudinal studies tracking emotional changes over the entire course of teacher education programs would provide a more comprehensive understanding of how emotions evolve over time. Moreover, employing a mixed-methods approach that includes objective measures such as physiological indicators of stress and emotion could complement self-reported data and offer a more nuanced view of students’ emotional experiences. Future research should also explore the impact of specific interventions designed to support emotional wellbeing in pre-service teachers, ultimately contributing to better educational outcomes and retention.

The study provides valuable insights into the emotional dynamics experienced by pre-service teachers during their first semester. Key findings revealed an initial prevalence of negative emotions that transitioned to positive emotions, largely due to supportive teachers and the autonomy of university life. This aligns with existing literature, while also presenting unique perspectives that highlight the importance of academic-related factors over personal and social ones. Practical implications suggest that educators and policymakers should enhance emotional support and improve administrative coordination to foster better student wellbeing and retention. Acknowledging limitations such as small sample size and reliance on self-reported data, future research should aim for more diverse samples and include objective measures to provide a comprehensive understanding of emotional trajectories. The study’s findings contribute significantly to the field by emphasizing the need for a holistic approach to teacher education that incorporates both cognitive and emotional support. This research accentuates the critical role of emotional wellbeing in educational settings and highlights the importance of targeted strategies to support pre-service teachers.

## Data Availability

The original contributions presented in the study are included in the article/supplementary material, further inquiries can be directed to the corresponding author.
